# ACCYolo: Transmission equipment inspection image detection method based on multi-scale and occluded targets

**DOI:** 10.1371/journal.pone.0335186

**Published:** 2025-10-28

**Authors:** Xi Chen, Fulong Yao, Rongbin Cui, Shulei Zhang, Haixing Li, Chunhe Song, Shimao Yu

**Affiliations:** 1 School of Software, Shenyang University of Technology, Shenyang, China; 2 School of Control Engineering, Northeastern University, Shenyang, China; 3 School of Artificial Intelligence, Shenyang University of Technology, Shenyang, China; 4 The Institute of Al for Industries, Nanjing, China; 5 Shenyang Institute of Automation, Chinese Academy of Sciences, Shenyang, China; King Fahd University of Petroleum & Minerals, SAUDI ARABIA

## Abstract

With the rising global demand for electricity, transmission infrastructure is becoming increasingly important as a key support for ensuring stable and reliable power supply.In recent years, UAVs have been widely used in the inspection and maintenance of transmission equipment due to their advantages of high efficiency, flexibility and intelligence, which have greatly improved the operation and maintenance efficiency and safety level.However, the transmission equipment itself is exposed to harsh natural environments during prolonged use, such as high temperatures, humidity changes, wind and sand erosion, as well as electromagnetic interference, coupled with complex topographical features, such as mountainous, hilly, and forested areas, which result in the transmission equipment inspection process being challenged by occlusion and large differences in dimensions.To cope with these problems, this paper proposes ACCYolo. a model based on the YOLOv10n architecture with the goal of improving image detection of transmission equipment under multi-scale and occluded targets in UAV-based scenes.On the one hand, the ACCYolo model, to solve the occlusion problem, incorporates the Acmix model, which incorporates the self-attention mechanism to achieve dynamic feature extraction, effectively improving the detection performance of the model in overlapping scenes.On the other hand, in order to cope with the size difference problem in multi-scale detection, the GELAN structure combines a lightweight design with the Programmable Gradient Information (PGI) mechanism to improve the accuracy of multi-scale target detection, while the ASFF module is designed to improve the accuracy of multi-scale target detection through adaptive spatial feature fusion.The experimental results show that. The proposed method shows significant advantages in transmission equipment monitoring tasks, Overall mAP@50 raise to 0.950, and provides an effective program to ensure the reliability of power supply.

## Introduction

In recent years, with the continuous promotion of the global “carbon neutral” [1] goal and the accelerated construction of new power systems, the power industry in all countries of the world is accelerating towards cleaner, more efficient and intelligent. As the key link between energy supply and demand, the safety, stability and economy of the transmission network have become core issues of widespread concern in the international community. In the face of large-scale access to new energy, interconnection of power grids across regions and increasingly complex operation and maintenance scenarios, the demand for accurate sensing and intelligent inspection of transmission equipment continues to grow globally. However, how to give full play to the advantages of UAV remote sensing images in the complex and changing field environment to realize efficient and reliable intelligent inspection is still an important technical bottleneck in the development of the current international smart grid.

The northeast region of Brazil has a very diverse climatic environment, consisting mainly of the vast Caatinga semi-arid zone in the interior and the humid coastal zone. The inland area is hot all year round, precipitation is scarce and spatial and temporal distribution is extremely uneven, frequently experiencing long-term drought and high temperature stress, is one of the world’s typical arid and vulnerable zone; the coast is affected by the Atlantic monsoon, precipitation is relatively abundant, but there are short-term heavy rainfall and occasional tropical storms and other extreme weather events. The high complexity and extremity of the regional climate pose a great challenge to the stable operation of transmission equipment and the adaptability of the intelligent inspection system.Therefore, in order to promote the research and application of intelligent inspection technology in extremely complex environments, it is urgently necessary to rely on highly diverse and authentic data sets to carry out method validation.

At present, the mainstream public power line datasets [2] include Power line dataset (2017), Tomaszewski et al. (2018), Tower dataset (2019), CPLID (2020), TTPLA (2020), STN PLAD (2021) and PLT-AI (2022). These datasets focus on conductors (such as Power line dataset, which only contains pixel-level segmentation of conductors), insulators (such as Tomaszewski et al. and CPLID, which are mainly used for target detection and are mostly single categories), transmission towers (Tower dataset, which only contains transmission tower targets), instance segmentation or target detection of multi-category components (such as TTPLA and STN PLAD), and PLT-AI, which contains a small number of defective components such as the Bird’s Nest. Although the above dataset utilizes UAV remote sensing imagery to provide strong data support for power asset identification and detection research, most of them have limitations such as limited asset categories, small number of annotations and samples, lack of defect types, and single task types, which makes it difficult to fully reflect the complexity and diversity of the real field power transmission environment.

To make up for the shortcomings of existing datasets, this paper uses the InsPLAD dataset collected by drones in real power transmission line scenarios in northeastern Brazil. InsPLAD covers 17 types of power assets, 28,933 component instances, 10,607 high-resolution images, and includes multiple types of real defective assets, which not only fully reflects the actual challenges of variable lighting, complex background, occlusion and natural equipment degradation in natural environment, but also supports various computer vision tasks such as target detection, image classification and anomaly detection. InsPLAD is significantly better than the above mainstream datasets in terms of asset category, sample size, defect type and task diversity, and is a representative and valuable test benchmark for the research and development of intelligent transmission equipment inspection methods and engineering applications.

Power transmission equipment is usually exposed to complex field environments for long periods of time [3], and is susceptible to a variety of factors such as wind and sand, drastic changes in temperature and humidity, mechanical fatigue and environmental corrosion, leading to degradation of its performance and even functional failure, which induces power line failures or wide-scale grid shutdown accidents. In order to reduce the risk of equipment failure, the traditional power system relies on manual inspection for periodic equipment monitoring. However, this method has significant problems such as low efficiency, high labor cost, and high security risk, which makes it difficult to meet the current operation and maintenance needs of “large-scale, high-density, and high-reliability” transmission networks.

At the policy level [4], developed and developing countries show significant stratification in the path of promoting smart grid policies. Developed countries such as the United States, Japan, and South Korea generally incorporate smart grids into their national energy strategies or legislative systems, and issue special bills and roadmaps to clarify phased construction goals and innovation orientations. These countries attach great importance to the construction of standard systems, information security and interoperability, promote the large-scale application of smart meters, advanced metering infrastructure (AMI), demand response and distributed renewable energy, and promote the deep integration of ICT and power systems through pilot demonstrations and market-oriented mechanisms. In contrast, developing countries, represented by mainland China, pay more attention to leading the upgrading of power grid infrastructure through national development planning, take the smart grid as an important hand in realizing energy transformation and grid modernization, emphasize the breakthroughs in core technology, the construction of independent intellectual property rights system and the synergy of the whole industrial chain, and promote the intelligent coverage of urban and rural power grids in a phased and coordinated manner. Overall, developed countries focus on innovation-driven and green and low-carbon integration, while developing countries emphasize planning-driven and localized innovation. The two complement each other and jointly promote the system upgrade and energy transformation of the global smart grid.

With the development of artificial intelligence, especially deep learning technology [5], image-based automated transmission equipment inspection has become an important means to realize intelligent perception. By means of high-resolution image acquisition [6] (e.g., drones, tower camera systems), combined with target detection algorithms, remote sensing, defect identification and structural analysis of transmission equipment status can be realized. However, there are still two prominent difficulties in practical applications: first, the types of power transmission equipment are diverse [7], and the structural sizes vary significantly [8]. Traditional detection models are difficult to balance the recognition accuracy of small and large targets; Secondly, interference factors such as occlusion and background complexity often exist in field environments, resulting in weak expression of equipment target features and high false detection and leakage rate [9], which seriously restricts the model’s generalization ability and engineering practicability.

Aiming at the above problems, this paper proposes transmission equipment inspection image detection based on multi-scale and occlusion targets based on the improved YOLO algorithm, aiming to build a deep detection framework that takes into account accuracy, speed and robustness, thereby effectively reducing operation and maintenance costs, improving detection efficiency and engineering feasibility. Specifically: (1) The Acmix module is introduced to fuse the self-attention mechanism with convolution to achieve dynamic regulation of feature extraction, significantly improving the model’s perception ability in scenarios where devices overlap or are blocked; (2) Design a lightweight GELAN structure in the backbone network and enhance the model computing efficiency through PGI mechanism to improve the scale detection efficiency; (3) Construct ASFF module to realize multi-scale spatial feature fusion and enhance the robust detection ability of the model for different sizes of equipment targets.

Through comparative experiments and ablation analysis on complex power transmission scenario datasets, the proposed method significantly improves the detection accuracy under multi-scale and occluded target conditions while maintaining the lightweight model, showing good engineering applicability and deployment value. This study not only provides key algorithm support for the new intelligent inspection system, but also provides a technical path reference for the intelligent transformation of my country’s power grid operation and maintenance model. The main contributions of this paper are as follows:

1. First, for the occlusion problem in the transmission equipment detection task, this paper improves the Neck. The improved Neck module has the ability of global sensing, and also captures local features through convolution, thus improving the performance of the model while maintaining a low computational cost.

2. In addition, in order to cope with the challenges of target detection of different sizes, this paper improves the Backbone and Head modules. The improved Backbone module, designs a lightweight GELAN structure in the backbone network, and enhances the model computing efficiency through the PGI mechanism to improve the efficiency of scale detection. The improved Head module introduces an adaptive spatial feature fusion approach, which enhances the scale invariance.

This paper is organized as follows. First, we introduce relevant prior work. Next, we present the proposed algorithm in detail. Then, we present experimental results and analyze them. Finally, we draw conclusions.

## Related work

In the power system, especially in the inspection of high-voltage transmission lines, drone inspections are widely used because of their high safety and efficiency. However, due to the characteristics of the transmission equipment itself and its complex operating environment, the inspection process faces challenges such as occlusion and large size differences. To address these issues, transmission equipment inspection image detection based on multi-scale and occluded targets is proposed. This will effectively reduce the burden of manual inspections, improve the accuracy of detection, and help staff complete inspection tasks more easily and efficiently.To facilitate our research, we have introduced Table 1 for analyzing relevant work. Each subsection below will analyze the table.

**Table 1:** Detection methods analysis.

Method Category	Representative Methods	Core Ideas	Obstruc-tion issue	Multi-scale Problem	Limitations/ Characteristics
	Haar + Cascade [10,12,13]	Manual features (Haar, HOG) + classifier detection	No	No	Limited feature expression, difficult to handle complex scenes
Traditional methods	SVM + ANFIS [11]	S-transform feature extraction + fuzzy reasoning recognition	No	No	Limited accuracy, relies on manual features
	SSD [14]	Single-stage anchor prediction, multi-layer feature detection	No	Yes	Insufficient target detection accuracy
Deep learning methods	YOLO [15]	Regression modeling, real-time detection	No	Partial	Occlusion sensitive, limited multi-scale robustness
	R-CNN series [16–18]	Region proposal + CNN feature extraction	No	Partial	Slow inference speed, not suitable for real-time
	Improved YOLOv8 (C2f_Res2block, MHSA, EMA) [19]	Attention mechanism + multi-scale feature enhancement	No	Yes	Strong scene specificity, insufficient generalization
	CSB-YOLO [20]	BiFPN multi-scale fusion + SCConv	No	Yes	Lightweight sacrifices some accuracy
	Improved SSD [21]	FPN fusion convolution and upsampling features	No	Yes	Insufficient performance under occlusion scenes
Improved Method	Improved YOLOv5 (EIOU, AFK-MC2, Cluster-NMS) [22]	New loss function + improved NMS	Yes	Partial	Increased algorithm complexity
	Improved YOLOv4 (Repulsion Loss etc.) [23]	Repulsion loss alleviates occlusion interference	Yes	No	Reduced inference efficiency
	RSA-YOLO [24]	Scale/ratio perception mechanism + intelligent segmentation	No	Yes	Sensitive to input image ratio
	TOD-YOLOv7 [25]	Small target detection layer + coordinate attention	Partial	Yes	Insufficient generalization ability
	UAV-YOLOv8 [27]	WIoU v3 + BiFormer + FasterNet fusion	No	Yes	Suitable for resource-constrained devices
	YOLO-SE [28]	SEConv + EMA + Transformer Head	No	Yes	Insufficient adaptability to complex backgrounds
	YOLO_AEF [30]	AFFM MEEM	No	Yes	Weak occlusion adaptability
**Methodology of This Paper**	**ACCYolo**	**YOLOv10 framework + ACmix (occlusion awareness), GELAN+PGI (multi-scale modeling), ASFF (adaptive feature fusion)**	**Yes**	**Yes**	**Simultaneously improves occlusion and multi-scale detection performance, maintains lightweight, suitable for power line inspection**

### Target detection model based on traditional methods

In the early stages of computer vision, target detection mainly relies on the combination of manual feature extraction and traditional classifiers, which are widely used in a variety of scenes. Early studies mostly used traditional methods based on the combination of Haar features and cascade classifiers. Specifically, Haar features scan the image block by block through a sliding window and combine with a pre-trained Haar cascade classifier to accomplish classification and detection. Zhao et al. [10] proposed to retain insulators based on orientation angle detection and a priori knowledge of the insulator’s binary shape and localize them with a minimum outer rectangular frame. By traversing all possible orientation angles, multiple insulators with different orientation angles can be localized. Jaya Bharata Reddy et al. [11] proposed the use of Discrete Orthogonal S-transform to extract image features and combined with Support Vector Machine (SVM) and Adaptive Neuro Fuzzy Inference System (ANFIS) to estimate the insulator condition and combined with Support Vector Machine classification to identify the state of the insulator. Zuo et al. [12] proposed a classifier that can recognize and locate insulators obtained by feature extraction and training with Haar features, integral map, cascade classifier, and directional gradient histogram, then segment the insulators by a series of digital image processing methods, and finally the insulator pixels obtained by segmentation are statistically analyzed to determine whether the insulators are missing or not. In order to improve the robustness of the model in such environments, Schwegmann et al. [13] proposed a novel vessel detection method, which first performs an initial screening by a constant false alarm rate (FAR) pre-screening step, and then uses a cascading classifier based on Haar-like features for vessel identification. However, these methods provide a solid foundation for target detection, they require manual design of feature extractors, have limited feature expressiveness, limited algorithmic recognition accuracy, and are difficult to cope with complex scenarios.

### Deep learning based target detection method

With the development of deep learning, specific network structures for target detection have emerged, which are divided into two main categories: single-stage detectors and two-stage detectors.

The single-stage detector directly predicts the targets in the image and accomplishes the detection of target location and category. Liu et al. [14] proposed Single Shot MultiBox Detector (SSD), a single-step anchor-box based target detection algorithm, which is able to predict a set of default bounding boxes from feature maps at different scales, handle targets of different sizes, and predict both the category confidence and adjustment within each bounding box. Joseph Redmon [15] proposed You Only Look Once (YOLO), which treats target detection as a regression problem and directly predicts the bounding boxes and their category probabilities in an image, providing a basis for the optimization of the subsequent YOLO series of algorithms.

The two-stage detector divides target detection into two steps: candidate region generation and classification. Ross Girshick et al. [16] proposed the RCNN, which combines region proposals with deep convolutional neural network (CNN) features. The method generates candidate regions through unsupervised learning, extracts features using pre-trained CNNs, and ultimately classifies the target with an SVM classifier. Subsequently, Ross Girshick et al. [17] proposed Fast R-CNN based on RCNN, where candidate regions are mapped to a fixed-size feature map through ROI pooling layer, and the image needs only one forward propagation to extract features, and then a fully connected layer is used to complete the classification and regression tasks, which significantly improves the speed and efficiency of the RCNN. Ren et al. [18] further proposed the Regional Proposal Network (RPN) to share the convolutional features of the image with Fast R-CNN, which reduces the computational cost.

These network structures have achieved significant results in target detection. By training deep learning models, they are able to detect classroom behaviors and improve the accuracy and efficiency of detection. However, these general methods have some limitations in different scenes and object detection, and still need a lot of adjustment and optimization for specific scenes.

### Improved deep learning based target detection

The general target detection method has certain limitations, but through the improved method based on the basic algorithm, we can see its feasibility. Haiwei et al. [19] proposed an improved YOLOv8 classroom behavior detection model, which improves the effectiveness and accuracy of YOLOv8 in classroom behavior detection by combining Res2Net and YOLOv8 network modules, proposing the C2f_Res2block module, and introducing MHSA and EMA mechanisms. In addition, to further enhance the feature extraction effect of the model, Wenqi et al. [20] proposed the CSB-YOLO model, which enhances multi-scale feature fusion through bidirectional feature pyramid network (BiFPN), designs an efficient reparameterized detection head (ERD Head) to improve the inference speed, and introduces self-calibrating convolution (SCConv) to compensate for the loss of accuracy in the lightweight design.

Li et al. [21] utilized a feature pyramid network to fuse the features of the upsampling layer and the invariant scale convolutional layer, and retained the multi-scale feature layer extracted from the traditional SSD structure, which improved the accuracy of detection. Ding et al. [22] replaced IOU with EIOU to calculate the box loss and introduced Assumption-free K-MC2 (AFK-MC2) algorithm based on yolov5, and cluster non-maximum suppression (Cluster-NMS), to improve the leakage of detection due to occlusion. Henghuai et al. [23] proposed an improved YOLO-v4 behavior detection algorithm that combined a cross-stage local network and an embedded connection component to improve the recognition ability of student and teacher behaviors. In order to solve the problem of difficult recognition of student behavior in the case of occlusion, the Repulsion loss function is added to reduce the misdetection and omission through RepGT and RepBox loss. Hsu et al. [24] proposed the RSA-YOLO (Ratio and Scale Aware YOLO) method, which aims to solve the problem of poor detection performance in pedestrian detection due to the small target scale and the large difference in the aspect ratio of the input image. The method dynamically adjusts the input layer hyperparameters of YOLOv3 by introducing a scale-aware mechanism, and adopts intelligent image segmentation technology to improve the detection accuracy. Meanwhile, a scale-aware mechanism of multi-resolution fusion is proposed to effectively improve the detection of pedestrians with small targets.

Tang et al. [25] proposed a YOLOv7-based target detection method, called TOD-YOLOv7, to address the challenges of low-resolution images, dense occlusions, and different poses in the TinyPerson dataset. By adding a small target detection layer to the YOLOv7 network, introducing a recursive gated convolution module and a coordinate attention mechanism, the model’s detection capability is enhanced and inference time is reduced. In addition, data enhancement techniques are combined to improve the algorithm’s representation learning capability. Wu et al. [26] proposed a Faster R-CNN-based Different Scale Face Detector (DSFD), which aims to solve the challenge of small-scale face detection. The method first obtains face ROIs through a multi-task regional proposal network (RPN) combined with augmented face detection, and assigns the proposals to three corresponding Fast R-CNN networks based on different proposal scales. Wang et al. [27] proposed a UAV target detection model based on YOLOv8 optimization, called UAV-YOLOv8, aiming to solve the problem of low accuracy and resource constraints in detecting small targets in UAV images. The model introduces Wise-IoU (WIoU) v3 as a regression loss function to improve the localization ability and optimizes the backbone network through BiFormer attention mechanism. A Focal FasterNet module is also designed for multi-scale feature fusion to improve the detection performance of small targets.

Tianyong et al. [28] proposed an improved YOLOv8 network called YOLO-SE, which aims to address the challenges of multi-scale target detection and small target detection in remote sensing images. By introducing lightweight SEConv convolution and SEF modules, the network parameters are reduced and the detection speed is accelerated. Also, an efficient multi-scale attention (EMA) mechanism is integrated to enhance the feature extraction capability. The network also contains specialized tiny target detection heads and uses Transformer [29] prediction heads instead of the original detection heads. In addition, the Wise-IoU loss function is introduced to cope with the gradient problem for low-quality instances. Wang et al. [30] proposed an adaptive enhancement fusion framework based on YOLO (YOLO_AEF). This framework employs a multiple exposure enhancement module (MEEM) to improve image quality under complex lighting conditions and utilizes an adaptive feature fusion module (AFFM) to fuse raw and enhanced image features, thereby enhancing robustness and contextual expression. Concurrently, it incorporates a fusion detection module (FDM) to achieve robust occlusion detection.

Although generalized target detection methods have shown better results, there are still some challenges for the detection of transmission line components. For example, the detection and target overlapping of existing methods for small-sized targets such as Spacer can affect the effectiveness of the deep learning model, yet it is difficult to obtain a large and sufficient amount of data for training due to the difficulty of transmission line data acquisition. Therefore, the research in this paper focuses on solving the problems of overlapping targets and large size differences in transmission line component detection. By improving the training method of the deep learning model, the detection accuracy and robustness of transmission line components can be improved.

## Method

**Fig 1:**
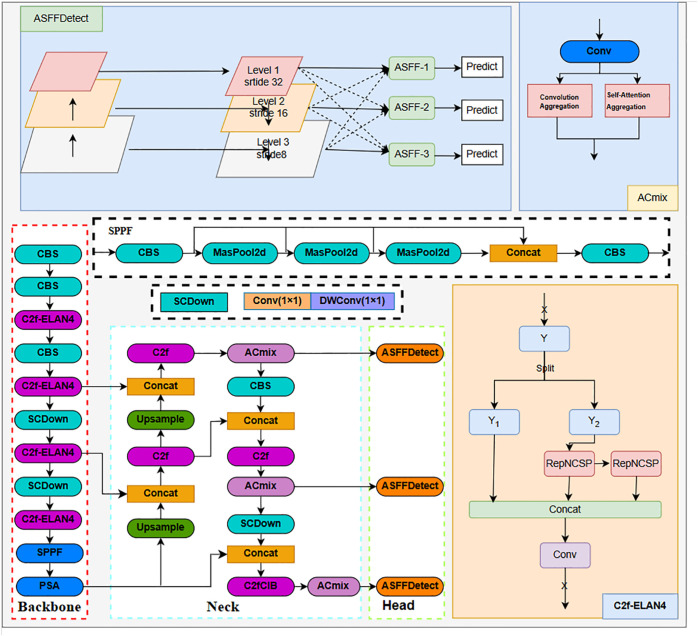
ACCYolo network structure diagram.

The main architecture of the model is shown in Fig 1. The backbone network of the architecture is yolov10. On this basis, the backbone module replaces the original C2f with C2f-ELAN4. For the Neck architecture, the ACmix attention mechanism is added to the output positions of the large, medium, and small target detection layers. The original v10Detect detection header is replaced with the ASFFDetect detection header in the Head. In the rest of this section, the ACmix attention mechanism, the ASFF detection header, and the GELAN module are described in detail for each of them.

### Improved neck module

In target detection tasks, the occurrence of occlusion phenomenon leads to the loss of local information about the target, which poses a challenge to the recognition performance of the model. The traditional Neck module, despite its self-attention mechanism, fails to effectively capture the contextual information of occluded objects due to its high computational complexity, leading to its unsatisfactory performance in dealing with occlusion situations. In order to counteract this problem, we have made improvements to the Neck module. Specifically, the ACmix module [31] was added after the output channels of the C2f and C2fCIB. With ACmix’s decomposition and reconstruction strategy, we are able to extract the contextual information of the occluded target more efficiently, Enhanced detection accuracy.

**Fig 2:**
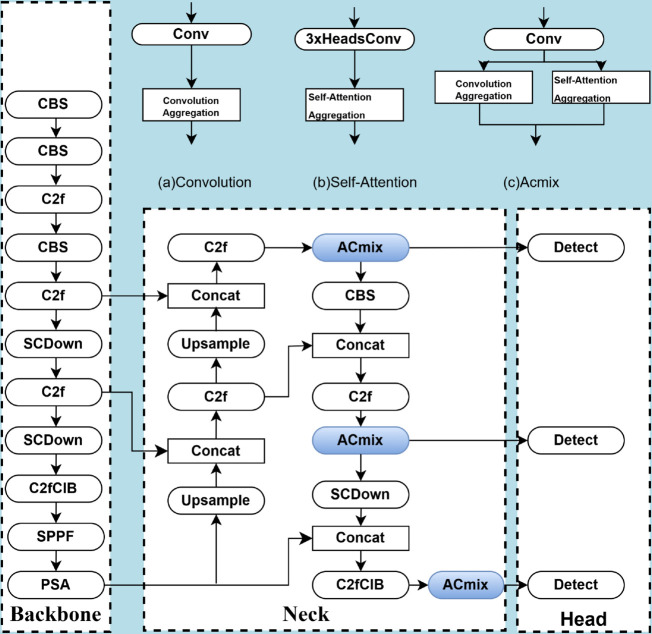
Architecture of the ACmix module.

This improvement leads to a significant optimization of the model’s performance in occlusion situations. ACmix combines the advantages of convolution and self-attention mechanisms (self-attention). Through analysis, both techniques operate similarly in the first stage, relying mainly on 1×1 convolution for feature projection, with computational complexity quadratically related to the number of channels. Based on this finding, ACmix shares convolutional and self-attentive projection operations, reducing computational overhead. While keeping the computational cost low, ACmix achieves significant performance gains on tasks such as image classification and target detection. Fig 2(a) and 2(b) represent the standard convolution and self-attention mechanisms, respectively. The workflow of the ACmix model is demonstrated in Fig 2(c).

ACmix forms a hybrid module by combining convolution and self-attention mechanisms. In the first stage, the input feature maps are projected through three convolutions to generate multiple intermediate feature maps. In the second stage, these intermediate feature maps follow two different aggregation methods: convolutional paths and self-attention paths.

For the self-attentive path, the generated intermediate feature maps are divided into groups and each group contains three feature maps which are used as query, key and value. Through the self-attention mechanism, the similarity between the query and the key is computed and the values are weighted and summed using the attention weights. This process can be represented by the following equation:

$$g_{ij}=\sum_{a,b\in N_{k}(i,j)}A(q_{ij},k_{ab})v_{ab}$$ (1)

where *q*_*ij*_ and *k*_*ab*_ are the feature representations of the query and key, respectively, $A(q_{ij},k_{ab})$ denote the attention weights, and $v_{ab}$ are the values.

For convolutional paths, a lightweight fully connected layer is used to generate *k*^2^ feature maps, which are shifted and summed by the following formula:

$$g_{ij}=\sum_{p,q}\text{Shift}(g_{ij}^{\left(p,q\right)},p,q)$$ (2)

Where $g_{ij}^{\left(p,q\right)}$ denotes the feature map obtained after applying the convolution kernel, and Shift(g,p,q) is a shift operation.

Eventually, the outputs from the convolutional and self-attentive paths are weighted and summed to form the final output. The formula is given below:

$$F_{\text{out}}=\alpha F_{\text{att}}+\beta F_{\text{conv}}$$ (3)

where *α* and *β* are learnable scalars that control the output strength of the self-attentive path and the convolutional path, respectively.

All figures and tables should be cited in the main text as Fig 1, Table 2, etc.

**Table 2:** Training parameter settings.

Parameters	Value
Training wheels	150
Batch size	18
Input Image Size	640×640
Optimizer	SGD
Initial learning rate	0.01
Momentum	0.937
Weight decay	0.0005
Warm-up rounds	3
Boundary box loss weights	7.5
Categorized loss weights	0.5
Distributed Focus Loss Weights	1.5
Verifying IoU Thresholds	0.7
Maximum number of tests	300
Data Enhancement Strategy	Mosaic, random flip (Level 0.5), color transformation (Hue 0.015, Saturation 0.7, Brightness 0.4)
Early Stop Mechanism	100 epoch Early stop without significant improvement
Mixed precision training	TRUE

### Improved backbone and head modules

**Fig 3:**
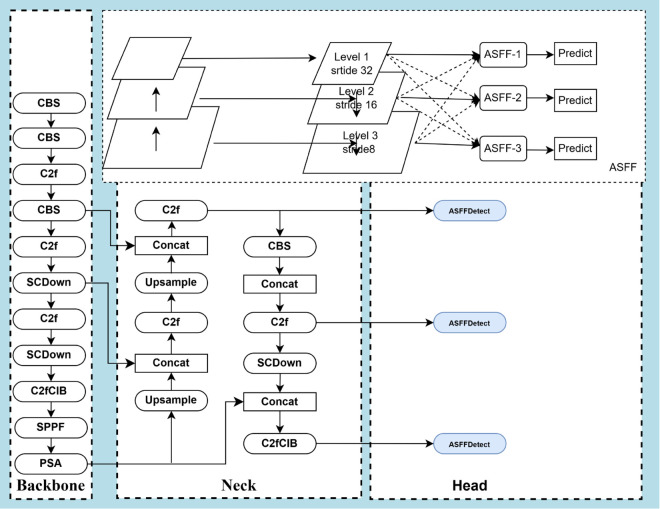
Schematic diagram of the ASFF module.

The improved Backbone module adds C2f-ELAN4 (GELAN) [32] to the output channel of CBS, GELAN incorporates CSPNet and ELAN mechanisms and utilizes RepConv to obtain more effective features while specializing in single-branching structure in inference, thus increasing the detection accuracy at multiple scales. The GELAN module improves feature extraction by combining multiple convolutional operations. As in Fig 3. first, the input feature map undergoes an initial convolution operation to generate an output feature map with channels, expressed as:

Y=Conv(X) (4)

where $Y\in {\mathbb{R}}^{H\times W\times C}$, *H* and *W* are the height and width of the feature map, respectively, and *C* is the number of channels. Next, the generated feature map *Y* is equally divided into two parts *Y*_1_ and *Y*_2_ with $\frac{C}{2}$ channels each, which can be expressed as:

$$Y_{1},Y_{2}=\text{Split}(Y)$$ (5)

where $Y_{1},Y_{2}\in {\mathbb{R}}^{H\times W\times \frac{C}{2}}$, the segmented feature map *Y*_2_ is subsequently processed through the RepNCSP module. The core of RepNCSP is a convolution operation combining 3×3 and 1×1. Eq:

$$Z_{i}=\text{RepNCSP}(Y_{i})={\text{Conv}}_{3\times 3}(Y_{i})+{\text{Conv}}_{1\times 1}(Y_{i})$$ (6)

where, i=2,3 this design allows the model to simultaneously process features from different receptive fields, enhancing the ability to characterize complex scenes.

Next, the two RepNCSP-processed features *Z*_2_, *Z*_3_ and *Y*_1_ are re-fused by a splicing operation Concat to form a complete feature map *Z*, denoted as:

$$Z=\text{Concat}(Y_{1},Z_{2},Z_{3})$$ (7)

where $Z\in {\mathbb{R}}^{H\times W\times C}$. Finally, the fused feature map *Z* is again subjected to a convolution operation to generate the final output $X_{\text{out}}$, Eq:

$$X_{\text{out}}=\text{Conv}(Z)$$ (8)

The design improves the expressive power of the model in feature extraction by separating the channels, feature processing with different convolutional kernels and the final fusion convolution operation, which is particularly suitable for tasks such as target detection where multi-scale features need to be processed.

**Fig 4:**
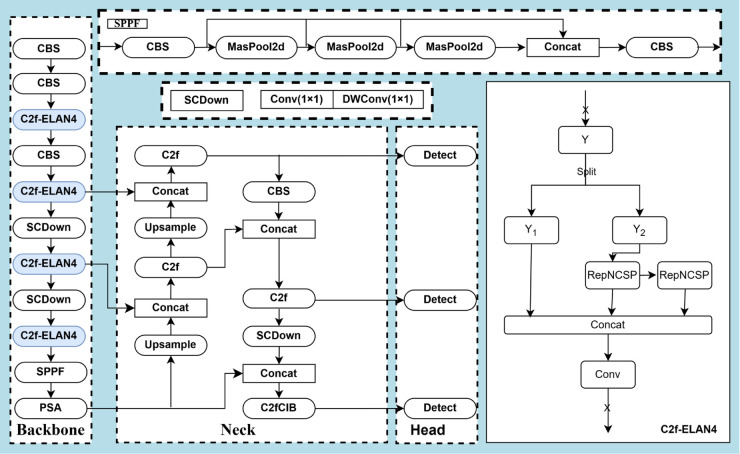
Schematic diagram of the GELAN architecture.

The improved Head module adds ASFF [33] to the output channels of ACmix, which introduces an adaptive spatial feature fusion method that effectively filters out conflicting information and thus enhances scale invariance. The proposed ASFF (Adaptively Spatial Feature Fusion) module aims to improve multi-scale target detection by fusing feature maps from different scales. Fig 4 illustrates the feature maps from different levels of the feature pyramid (Level 1, Level 2, Level 3) with different resolutions and strides. The ASFF module adjusts the weighting of the features at each scale using the adaptive weights $\left(\alpha^{3},\beta^{3},\gamma^{3}\right)$, which are combined with the feature maps of each level (X1 → 3, X2 → 3, X3 → 3) by element-by-element multiplication, ensuring that the contributions of each level can be effectively integrated. to ensure that the feature contributions of each layer can be effectively integrated. Especially for multiscale detection, low-level high-resolution features are crucial, and the ASFF module generates unified features for subsequent target prediction by adaptively fusing different scale features, and, finally, fusing the scale features after an additive operation.

## Experiments

The training parameters of the experiment based on the YOLOv10 model for the transmission equipment detection task are shown in Table 2 to explore the performance of the model in complex scenarios. First, the configuration files of the model and data were loaded and key training parameters were set, including the number of training rounds, batch size, learning rate and momentum. In the data preprocessing stage, data enhancement techniques such as Mosaic, Random Flip and Color Transform (including random adjustments of hue, saturation and luminance) are used with the aim of improving the generalization ability of the model and reducing possible overfitting phenomena. In addition, pre-training weights are used for model initialization to accelerate model convergence. During the training process, SGD is used as an optimizer with a warm-up phase and a dynamic learning rate adjustment strategy to improve the convergence speed of the model. After each round of training, metrics including precision, recall, mAP@50 and mAP@50-95 evaluations are computed on the validation set to monitor the performance of the model in the transmission equipment detection task in real time. In order to avoid overfitting, an early stopping mechanism is set up, and the training is automatically terminated early when no significant performance improvement occurs within 100 epochs. This experiment generates graphs of loss and performance metrics during training and validation to provide reliable data support for model optimization and performance improvement.

### Experimental setup and data description

The code for the paper was implemented on a server equipped with 2 RTX4090 GPUs. As shown in Table 3, compared to earlier datasets such as Power line dataset (2017) and Tomaszewski et al. (2018) that contain only a single asset class and lack defective samples, the InsPLAD dataset has a significant lead in terms of the number of asset classes (17), the number of annotations (28,933) and the number of images (10,607), and covers 5 classes of defective assets. significantly ahead of the others and covers 5 classes of defective assets, supporting a variety of visual tasks such as Object Detection (OD), Image Classification (IC) and Anomaly Detection (AD). Therefore, the dataset in this paper adopts InsPLAD, a dataset and benchmark for power line asset inspection, with specific parameters shown in Table 4.

**Table 3:** Public power line datasets.

Dataset	Power line asset classes	Annotations	Images	Defective asset classes	Vision tasks
Power line dataset (2017)	1	4,200	4,200	0	SS
Tomaszewski et al. (2018)	1	2,630	2,630	0	OD
Tower dataset (2019)	1	Undisclosed	1,300	0	OD
CPLID (2020)	1	1,569	848	1	OD
TTPLA (2020)	2	8,987	1,100	0	IS
STN PLAD (2021)	5	2,409	133	0	OD
PLT-AI (2022)	5	17,808	6,295	4	OD
**InsPLAD**	**17**	**28,933**	**10,607**	**5**	**OD, IC & AD**

**Table 4:** Basic parameters of the InsPLAD dataset.

Asset category	Number of images	Label
Damper-Spiral	943	1020
Damper-Stockbridge	1761	6953
Glass Insulator	2778	2978
Glass Insulator Big Shackle	152	259
Glass Insulator Small Shackle	143	263
Glass Insulator Tower Shackle	106	195
Lightning Rod Shackle	112	195
Lightning Rod Suspension	709	710
Tower ID Plate	242	242
Polymer Insulator	3173	3244
Polymer Insulator Lower Shackle	1760	1842
Polymer Insulator Upper Shackle	1691	1692
Polymer Insulator Tower Shackle	57	57
Spacer	93	94
Vari-grip	560	1008
Yoke	1661	1661
Yoke Suspension	2716	6520

### Ablation experiment

In Table 5, we show the results of the ablation experiments of the model by systematically investigating the independent contribution of each module to the model performance in order to assess its importance in complex detection tasks. In this set of experiments, experiments 0, 1, 2, 3, 4, 5, 6 and 7 were tested on raw data. Labeled A(Acmix), B(ASFF), C(GELAN), where a0, a1, a2 are for multi-scale target detection and a3, a4, a5 are for occluded target detection.

**Table 5:** Ablation study results of YOLOv10 with different modules.

Class/Model	0	1	2	3	4	5	6	7
	YOLOv10	YOLOv10+	YOLOv10+	YOLOv10+	YOLOv10+	YOLOv10+	YOLOv10+	YOLOv10+
		GELAN	ASFF	Acmix	GELAN+ASFF	ASFF+Acmix	GELAN+Acmix	GELAN+ASFF + Acmix
yoke	0.959	0.953	0.950	0.968	0.969	0.955	0.962	**0.973**
yoke suspension	0.991	0.991	0.989	0.991	0.991	0.990	0.990	**0.992**
spacer	0.631	0.845	0.871	0.869	0.862	0.770	0.839	**0.967**
stockbridge damper	0.982	0.984	0.978	0.982	0.985	0.982	0.978	**0.988**
lightning rod shackle	0.713	0.747	0.811	0.719	0.821	0.868	0.814	**0.924**
lightning rod suspension	0.995	0.995	0.994	0.995	0.995	0.995	0.994	**0.995**
polymer insulator	0.990	0.992	0.992	0.990	0.992	0.988	0.987	**0.989**
glass insulator	0.958	0.968	0.959	0.971	0.973	0.946	0.959	**0.991**
tower id plate	0.995	0.995	0.995	0.995	0.995	0.995	0.995	0.987
vari-grip	0.993	0.992	0.993	0.994	0.994	0.993	0.994	0.992
polymer insulator lower shackle	0.867	0.897	0.885	0.902	0.905	0.873	0.865	**0.963**
polymer insulator upper shackle	0.990	0.990	0.990	0.991	0.992	0.987	0.990	**0.993**
polymer insulator tower shackle	0.972	0.995	0.995	0.995	0.995	0.956	0.995	0.930
glass insulator big shackle	0.325	0.430	0.409	0.469	0.472	0.403	0.445	**0.830**
glass insulator small shackle	0.364	0.449	0.402	0.545	0.486	0.363	0.339	**0.805**
glass insulator tower shackle	0.522	0.640	0.545	0.613	0.619	0.438	0.476	**0.805**
spiral damper	0.995	0.992	0.995	0.995	0.995	0.991	0.995	0.987
**all classes**	**0.838**	**0.874**	**0.868**	**0.881**	**0.885**	**0.852**	**0.860**	**0.950**

First, in the base model without any improvement module (experiment a), the overall mAP@50 performance is 0.838. Due to the lack of specific module support, the base model has obvious deficiencies in coping with target occlusion and scale differences. For example, the low detection effect of Stockbridge Damper and Yoke Suspension indicates that the base model is limited in dealing with these complex detection tasks as shown by a(0) in Fig 2. The prediction of Stockbridge Damper reaches 0.7 and the prediction of Yoke Suspension reaches 0.45.

**Fig 5:**
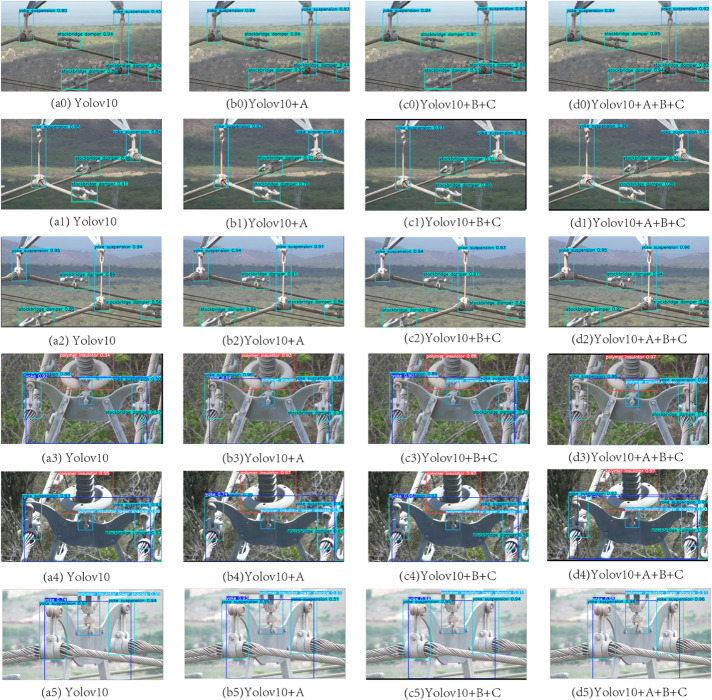
Detection results of YOLOv10 with progressively enhanced modules.

The experiment with the addition of the Acmix module (experiment d) shows that the detection effect is as shown in Fig 5, b(5), where the Polymer Insulator Tower Shackle detection efficiency is improved from 0.3 to 0.91 compared to the original a(5). b(4) in Fig 5 also shows that compared to the original a(4), the Polymer Insulator Tower Shackle detection efficiency is improved from 0.86 to 0.89 compared with the original a(4) in Fig 5, which effectively solves the target occlusion problem.

The experiment with the addition of GELAN and ASFF modules (experiment e), the detection effect is shown as b(1) in Fig 5, compared with the original a(1) Yoke Suspension, the detection effect is improved to 0.84 improved to 0.91, and Stockbridge Damper is improved from 0.41 to 0.89, respectively. improves the multi-scale complex background detection efficiency and effectively enhance multi-scale target detection.

Finally, the complete model containing all enhancement modules (experiment h) achieves the best performance on all categories, overall mAP@50 raised to 0.950. The synergy of the modules with the introduction of data enhancement strategies not only improves the detection performance of the model in multi-scale, complex background and occluded target environments, but also significantly enhances the model’s generalization ability.

### Comparison experiment

In order to verify the soundness of the proposed method, we compare it with several classical target detection methods, yolo11 [34], rtdetr-l [35], yolov8 [36], rtdetr-x [37], yolov9 [38], rtdetr-resnet50 [39], yolov10 [40], rtdetr-resnet101 [41]. As shown in Table 6. Our improved model ACC_yolov10_Enhance (ours) addresses the two key challenges of the transmission equipment inspection task (overlapping targets and large size difference) and significantly outperforms the other models in mAP@50. With the tabular data, we can clearly see how ACC_yolov10_Enhance (ours) compares with other models on different categories, further validating its advantages in complex detection tasks.

**Table 6:** Performance comparison of object detection models for transmission equipment inspection (mAP@50).

Class/Model	ACC_yolov10_ Enhance (ours)	yolo11	rtdetr-l	yolov8	rtdetr-x	yolov9	rtdetr- resnet50	yolov10/rtdetr-resnet101
yoke	**0.973**	0.955	0.929	0.963	0.943	0.964	0.915	0.953
yoke suspension	**0.992**	0.992	0.978	0.990	0.985	0.991	0.976	0.991
spacer	**0.967**	0.806	0.721	0.876	0.700	0.775	0.778	0.845
stockbridge damper	**0.988**	0.982	0.962	0.982	0.967	0.981	0.957	0.984
lightning rod shackle	**0.924**	0.794	0.722	0.820	0.856	0.908	0.781	0.747
lightning rod suspension	**0.995**	0.995	0.995	0.995	0.995	0.995	0.991	0.995
polymer insulator	**0.989**	0.990	0.975	0.991	0.981	0.993	0.963	0.992
glass insulator	**0.991**	0.970	0.943	0.965	0.933	0.978	0.927	0.968
tower id plate	0.987	**0.995**	0.995	0.995	0.995	0.995	0.994	0.995
vari-grip	0.992	0.994	0.992	0.994	0.993	0.994	0.990	0.992
polymer insulator lower shackle	**0.963**	0.907	0.828	0.910	0.862	0.928	0.844	0.897
polymer insulator upper shackle	0.993	0.992	0.979	0.992	0.988	0.993	0.972	0.990
polymer insulator tower shackle	0.930	**0.995**	0.696	0.995	0.926	0.995	0.831	0.995
glass insulator big shackle	**0.830**	0.580	0.385	0.531	0.414	0.571	0.380	0.430
glass insulator small shackle	**0.805**	0.560	0.388	0.518	0.353	0.584	0.380	0.449
glass insulator tower shackle	**0.805**	0.624	0.475	0.681	0.476	0.671	0.430	0.640
spiral damper	0.987	0.994	0.990	0.995	0.990	0.995	0.965	0.992
**all classes**	**0.950**	**0.890**	**0.821**	**0.894**	**0.845**	**0.901**	**0.828**	**0.874**

**Fig 6:**
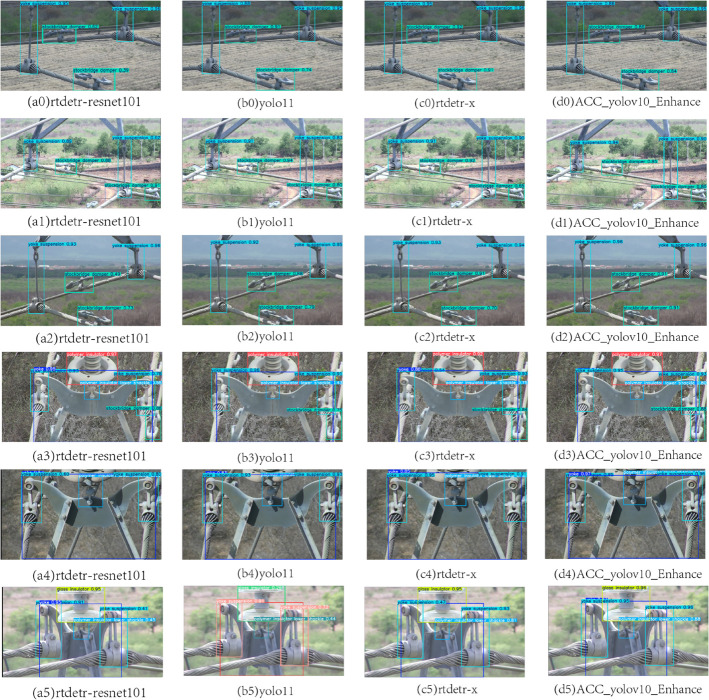
Comparative visualization of detection performance across different models (Ours vs. YOLOv8/RT-DETR).

In the “target occlusion” problem, ACC_yolov10_Enhance (ours) utilizes the Acmix module to combine the self-attention mechanism with convolutional feature extraction to improve the model’s discriminative ability in complex backgrounds. In the category of “lightning rod shackle”, the mAP@50 of ACC_yolov10_Enhance (ours) reaches 0.924, which is significantly higher than that of yolo11 (0.794) and yolov8 (0.82), and the mAP@50 of ACC_yolov10_Enhance (ours) is 0.924, which is significantly higher than that of yolo11 (0.794) and yolov8 (0.82). In the category of “Polymer Insulator Tower Shackle”, d(3) 0.75 in Fig 6 is improved to c(3) 0.8. The results show that the improved model has stronger detection accuracy in the scene with more serious target occlusion.

To address the problem of “large size difference”, ACC_yolov10_Enhance (ours) enhances the adaptability to multi-scale targets through the GELAN and ASFF modules. For example, in the category of “polymer insulator lower shackle”, which has a large size difference, although other models such as yolo11 and yolov9 also have high performance in this category (0.907 and 0.928, respectively), ACC_yolov10_Enhance (ours) is not able to adapt to multiscale targets through the GELAN and ASFF modules. yolov10_Enhance (ours) is more robust in the overall test, and in particular more balanced in terms of overall performance in the different categories. In addition, in the “yoke” category, ACC_yolov10_Enhance (ours) reaches 0.973, while yolov10 only reaches 0.953. In the “Yoke Suspension, Polymer Insulator Tower Shackle” category, ACC_yolov10_Enhance (ours) is more robust in the overall test, especially in the balanced performance of the different categories. Polymer Insulator Tower Shackle,” in the category of “Yoke Suspension,” d(5) 0.47,0.81 in Fig 6 improves to c(5) 0.95,0.88. This shows the excellent performance of the improved model on large size targets.

Combining the results of “all classes”, the overall mAP@50 of ACC_yolov10_Enhance (ours) reaches 0.95, which is significantly higher than that of yolov9, which is the best performer among other models (0.901). In the overall comparison of different categories, ACC_yolov10_Enhance (ours) shows high stability and accuracy under all kinds of challenges, providing higher reliability and accuracy for the automated inspection of transmission equipment.

**Fig 7:**
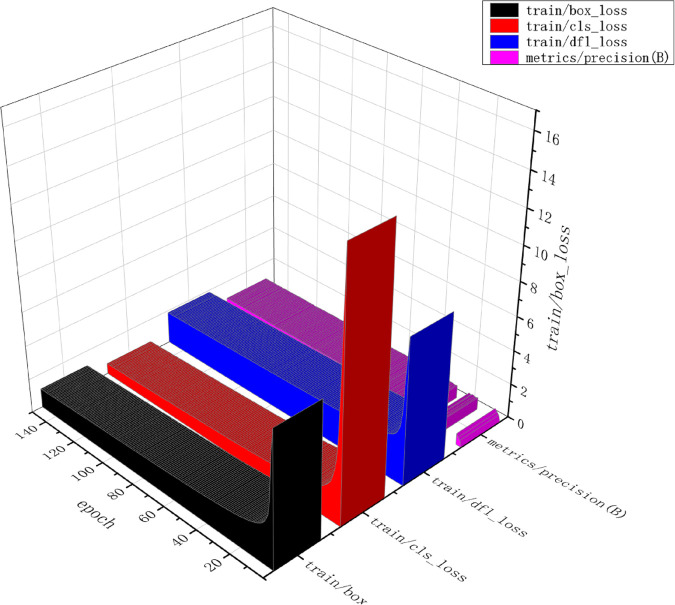
Convergence trends of training loss components (Box/Cls/DFL).

**Fig 8:**
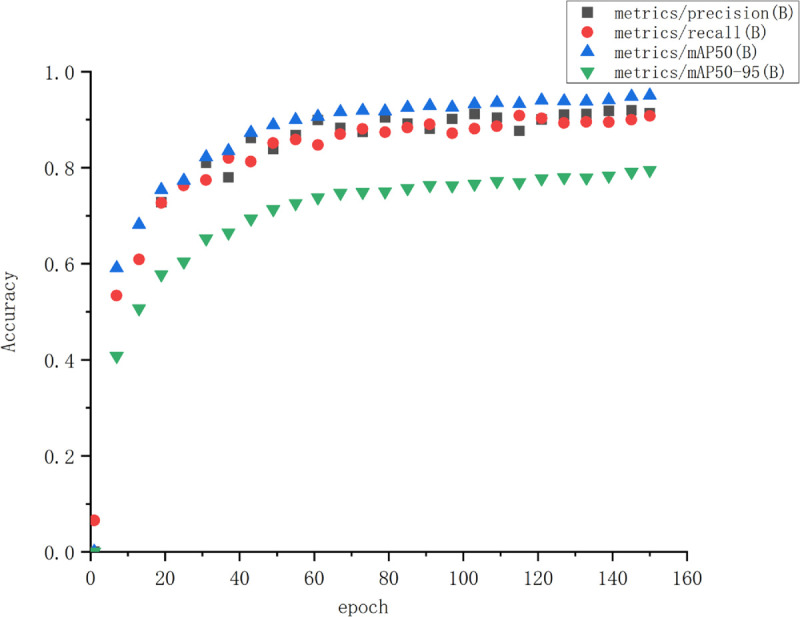
Evolution of model performance metrics during training (Precision/Recall/mAP).

**Fig 9:**
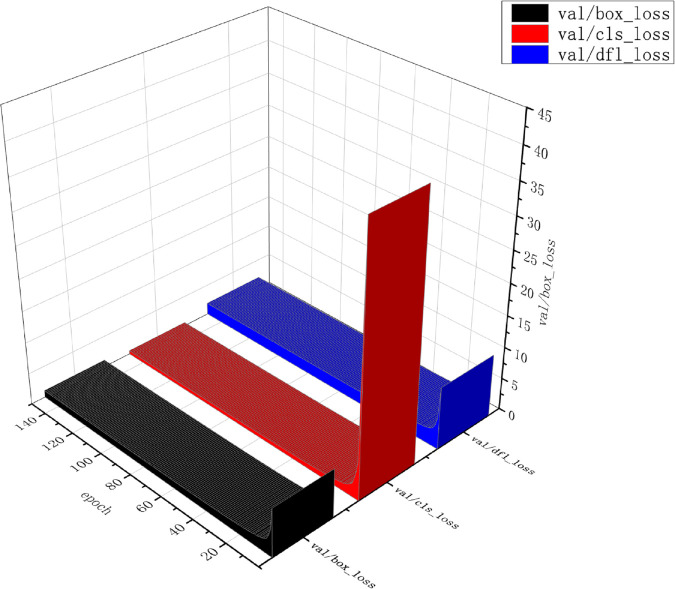
Validation loss analysis across training epochs (Box/Cls/DFL).

Figs 7, 8, and 9 demonstrate the trends of key loss terms and evaluation metrics for the ACC_yolov10_Enhance model over 150 training cycles. Specifically, the bounding box loss (box loss), classification loss (cls loss), and distribution focus loss (DFL loss) on the training and validation sets decrease rapidly at the initial stage, and then level off, indicating that the model converges gradually. In addition, the performance metrics such as precision, recall, and average precision (mAP@50 and mAP@50-95) increase rapidly in the early stage and stabilize at a high level in the later stage, reflecting the gradual improvement of the model’s performance on the detection task and its good generalization ability. Overall, the figure shows that the model achieves good convergence during training and stable performance on the validation set.

## Conclusion

In this study, an ACCYolo model is designed. An innovative solution is proposed to address the key challenges in target detection in complex backgrounds, such as target occlusion, size difference and background complexity. By introducing the Acmix model and combining the self-attention mechanism with the convolutional mechanism, the detection ability of the model in target occlusion and overlapping scenes is significantly improved. Meanwhile, the combination of the GELAN structure and the programmable gradient information (PGI) mechanism makes the model more efficient in dealing with multi-scale targets and improves the detection speed while ensuring the accuracy. In addition, the ASFF module further improves the accuracy of multi-scale target detection through adaptive spatial feature fusion, and shows strong robustness especially in complex backgrounds.

In order to verify the effectiveness of the proposed method, we conducted ablation experiments and comparison experiments. The ablation experiments show that the introduction of the Acmix module effectively solves the target occlusion problem and significantly improves the detection accuracy. For example, the detection accuracy of the Polymer Insulator Tower Shackle category is improved from 0.3 to 0.91, and the addition of the GELAN and ASFF modules also significantly improves the detection accuracy, especially in the multi-scale target detection task, the detection accuracy of the Stockbridge Damper and Yoke Suspension categories improved from 0.41 and 0.84 to 0.89 and 0.91, respectively.

When compared with classical target detection methods (e.g., YOLOv10, YOLOv8, RTDETR, etc.), the present method demonstrates significant advantages. In the category of “Lightning rod shackle”, the ACC_yolov10_Enhance (the present method) mAP@50 reaches 0.924, which is much higher than 0.794 for YOLOv11 and 0.82 for YOLOv8. Meanwhile, in the category of “Polymer Insulator Tower Shackle”, the detection accuracy is improved from 0.75 to 0.8, which shows the powerful detection ability of this method in complex background.

The combined detection results of all categories, ACC_yolov10_Enhance has an overall mAP@50 of 0.95, are significantly higher than the best performance of other comparison models (0.901 for YOLOv9). This indicates that the method proposed in this study not only has obvious advantages in detection accuracy, but more importantly, it effectively reduces the cost of transmission line inspection and greatly improves work efficiency by replacing the traditional manual inspection with automation technology. Before the improvement, the model’s frame rate was 7.58 FPS (frames per second). After the improvement, the frame rate increased to 15.13 FPS, demonstrating a significant enhancement in real-time processing capabilities. Compared with the traditional manual inspection, the automated inspection system is able to monitor the equipment 24 hours a day, discover potential problems in real time, avoid the expansion of faults, and reduce the investment of manpower and resources, thus improving the inspection efficiency while reducing the economic losses.

The method in this study is not only in the power system, but also has strong versatility and can be widely extended to other industrial inspection fields, such as health monitoring of transportation facilities, bridge structures and other critical infrastructures. These fields are generally characterized by technical difficulties such as target occlusion and large differences in the scale of the detection object, etc. In the future, this technology will provide solid technical support for the stable operation of the smart grid and promote the development of the power system in the direction of intelligence, providing an effective guarantee for the reliability of the global power supply.
